# Current situation, strengths and problems in intra- and interprofessional collaboration in German nursing homes – A holistic multiple case study

**DOI:** 10.1186/s12877-024-05182-z

**Published:** 2024-07-17

**Authors:** Kathrin Schmüdderich, Jonas Dörner, Anne Fahsold, Rebecca Palm, Martina Roes, Bernhard Holle

**Affiliations:** 1 Deutsches Zentrum für Neurodegenerative Erkrankungen (DZNE), Stockumer Str. 12, 58453 Witten, Germany; 2 Faculty of Health, School of Nursing Science, Witten/Herdecke University, Alfred-Herrhausen-Straße 50, 58455 Witten, Germany; 3 School VI Medicine and Health Sciences, Department of Health Services Research, Carl von Ossietzky University Oldenburg, Ammerländer Heerstraße 114-118, 26129 Oldenburg, Germany

**Keywords:** Professional collaboration, Professional relations, Organization of care, Role development, Residential facilities, Qualitative research, Case studies

## Abstract

**Background:**

The increasing care complexity of nursing home residents living with dementia requires new care models that strengthen professional collaboration. To contribute to the sustainable implementation of new care models, it is important that they are linked to the care reality. However, little is known about intra- and interprofessional organization and provision of care in German nursing homes. Therefore, the aim of this study was to explore the current care situation, problems and strengths regarding intra- and interprofessional collaboration in the care of residents living with dementia.

**Methods:**

We conducted a holistic multiple case study. The individual care units in which residents living with dementia are cared for were defined as cases. The context was built by the respective nursing homes and their regional affiliation to the federal state of North Rhine-Westphalia. We used qualitative face-to-face interviews, documents and context questionnaires for data collection. The different sources of evidence served to capture complementary perspectives and to validate the findings. First, the collected qualitative data were analyzed using deductive-inductive content analysis. Second, similarities and differences between the cases were identified to elaborate case-specific and cross-case patterns and themes. The reporting followed the EQUATOR reporting guideline for organizational case studies.

**Results:**

We included four care units comprising 21 professionals (nurses, physicians, social worker, physiotherapist, pharmacist) and 14 relatives of residents living with dementia. The analysis revealed four categories to describe current intra- and interprofessional collaboration in all cases: *actors and their roles, service delivery, coordination and governance,* and *communication channel*. Moreover, we identified three categories that relate to the strengths and problems of intra- and interprofessional collaboration in all cases: *role understanding, teamwork,* and *communication and exchange*. Although we examined similar care units, we found differences in the realization of professional collaboration and resulting problems and strengths that are connected to the organizational contexts and strategies used.

**Conclusions:**

Even though professional collaboration follows given patterns; these patterns do differ context-specifically and are perceived as problematic and fragmentary. Therefore, the identified differences and problems in collaboration need to be addressed in future research to develop and successfully implement tailored innovative care models.

**Supplementary Information:**

The online version contains supplementary material available at 10.1186/s12877-024-05182-z.

## Background

Due to demographic developments, the number of people in need of care and people living with dementia will increase in the coming years in Europe [1, 2]. As already a high number of nursing home residents in Germany are living with dementia and multimorbidity [2, 3], this development will further intensify the complexity of care in nursing homes. To address this complexity, the Federal Ministry of Health in Germany has recommended the implementation of innovative nurse-led care models. In these models, highly qualified nurses ought be deployed with new activities and roles according to their competencies, and interprofessional collaboration should be promoted to ensure high-quality, effective and efficient care [4]. Further, a new law will allow academically trained nurses to independently take on care and therapeutic activities for selected patient groups (e.g., people living with dementia) or specific health problems (e.g., chronic wounds) from 2025 onward. This means that some activities previously reserved for physicians can now be conducted autonomously by academically trained nurses with the required competencies (BGBl. 2023 I No. 359).

A recent scoping review on nurse-led care models in nursing homes showed that there are no nurse-led care models for German nursing homes developed yet, but a few that exist internationally [5]. These models could improve resident-, staff- and process-related outcomes and advance nursing roles. Further, they share many core elements for care coordination suggested from the SELFIE (Sustainable intEgrated chronic care modeLs for multi-morbidity: delivery, FInancing, and performancE) framework [5, 6]. Nonetheless, they also show several differences, which are related not only to the addressed resident-, staff- and process-related outcomes but also to the realization of intra- and interprofessional collaboration [5]. Therefore, to select relevant outcomes and elements for a German nurse-led care model and to address the general conditions of the German health care context, it seems necessary to obtain profound insight into intra- and interprofessional collaboration in German nursing homes.

Intraprofessional collaboration in nursing can be understood as “*a relational and respectful process among nursing colleagues that allows for the effective use of the knowledge, skills and talents of all nursing designations to achieve optimal client and health system outcomes*” [7]. In contrast, the World Health Organization (WHO) defines interprofessional collaboration in practice as “*health-care [that] occurs when multiple health workers from different professional backgrounds provide comprehensive services by working with patients, their families, carers and communities to deliver the highest quality of care across settings*” [8]. The general assumption in health care is that both intra- and interprofessional collaboration efforts lead to better health care services and outcomes for the respective populations [7–9]. Therefore, D’Amour defined key concepts that are relevant to collaboration in health care, including exchange, partnership, interdependence, and power [9]. An international review confirmed that interdisciplinary interventions have positive impacts on resident outcomes in nursing homes [10]. Similarly, a German study showed that an intervention for extended coordinated medical care in nursing homes could improve on-site medical care and the assessment of interprofessional collaboration [11].

In Germany, there is great heterogeneity in how care can be provided in nursing homes. In contrast to other countries, residents are for example free to choose their own physician. In many cases, residents therefore do not change the physician when moving into a nursing home [12]. Moreover, none of the physicians and only few of the therapists are generally employed in nursing homes, which means that a large number of collaborations with different people arise [2, 12, 13]. Nevertheless, the ways in which intra- and interprofessional collaboration takes place in nursing homes, especially in Germany, has been little explored thus far. Previous international studies in the nursing home setting have focused on single aspects, such as the organization of interdisciplinary palliative care for residents living with dementia and complex disabilities [14], perceptions of interprofessional collaboration in terms of role allocation and task description [15], and descriptions of the nature of information exchange in the interdisciplinary care of older people [16]. None of these studies have reported aspects of intraprofessional collaboration, although these insights could be of interest for researchers in other countries, who consider to develop and test new models of responsibility and task distribution in nursing care. In German studies, the focus of describing the organization and provision of collaboration has thus far been on interprofessional collaboration and task distribution between nurses and general practitioners (GPs) during nursing home visits [17–19]. As the German studies have neither included allied health professions other than nurses and GPs, e.g., social workers, therapists or medical specialists [17], nor focused on differences and similarities between organizations, there is still little knowledge about how professional collaboration is currently organized in German nursing homes. Nevertheless, such knowledge about the current care reality is relevant to determine the need for a new nurse-led care model [20]. The description of current care further allows us and researchers in other countries to decide which intervention aspects of intra- and interprofessional collaboration – compared to usual care – make an intervention successful and should be included in a new care model.

In this study, we focused specifically on the organization and provision of intra- and interprofessional collaboration in the care of residents living with dementia in German nursing homes, as our general aim of the overall project was to develop a dementia-specific nurse-led care model. Therefore, the aim of this study was to explore how intra- and interprofessional collaboration in the care of residents living with dementia in nursing homes is organized and provided and to investigate what problems and strengths in intra- and interprofessional collaboration can be identified. Herein, we focused on cross-context similarities and differences. The specific research questions addressed are as follows*:*

1. *How is the distribution, coordination and delivery of tasks, as well as the management of care of residents living with dementia, organized and provided within and between involved professional groups in German nursing homes?*
2. *What are perceived problems and strengths in the organization and provision of intra- and interprofessional collaboration in German nursing homes?*

## Methods

The reporting of this study followed the EQUATOR (Enhancing the Quality and Transparency Of health Research) reporting guideline for organizational case studies [21] (Additional file 1). No study protocol was registered.

### Design

We conducted a holistic multiple case study following Yin [22]. As there are few documented examples of the organization and provision of intra- and interprofessional collaboration in nursing homes and related problems and strengths, a case study design allowed for an in-depth empirical investigation of this complex phenomenon in a real-life context [22]. Thus, professional collaboration was examined taking into account the context from different perspectives to achieve a holistic understanding of the current situation.

We defined the single care units in which care of residents living with dementia takes place as cases. Thus, each case represented a real-world organization [22, 23]. The temporal boundary of the case was formed by the period of data collection (February to August 2022). The nursing homes and their affiliation to the federal state of North Rhine-Westphalia formed both the context of the cases and the spatial boundary. The purpose of defining multiple care units as cases was to obtain more robust findings and to be able to compare different organizational models. This allowed conclusions about relevant cross-context and context-specific aspects of intra- and interprofessional collaboration [22].

### Study setting and participants

Information about the study and criteria for participation was shared via e-mail with representatives of organizations, including nursing homes the DZNE in Witten collaborates with. The nursing homes that were interested in participating contacted us and took part in a Kick-Off event, where we provided more details about the study. Following Yin, four care units from different nursing homes were then selected to represent usual care in German nursing homes (typical cases) and to serve as a replication of the research question and method [22, 24]. Inclusion criteria for the care units were that they were located in the federal state of North Rhine-Westphalia. To exemplify usual care, care units with specific conceptual orientations were excluded (e.g., palliative care). Professionals and relatives of residents living with dementia were made aware of the study by the nursing home managers.

Regarding participants, we included professionals who were involved in the care and treatment of residents living with dementia in the included care units, who had at least one year of professional experience and who were trained in a field relevant to health care. This included nurses with three years of education and social workers from the nursing homes, as well as physicians, pharmacists and therapists, as external professionals. Additionally, we included relatives of residents living with dementia. Insufficient German language abilities or inadequate hearing ability for oral communication served as exclusion criteria for both groups. As previous studies have shown that residents can provide little information on professional collaboration [17], and that the questions could be even more abstract for residents living with dementia, we did not interview residents. Instead, resident files of four to five residents living with dementia were included per case. Inclusion criteria were a diagnosed dementia and a score above two in the Dementia Screening Scale (DSS) [25].

### Data collection

For an in-depth description and analysis of the cases we used different sources of evidence: qualitative interviews, postscripts, documents and context questionnaires [22, 24].

The first author conducted face-to-face problem-centered interviews [26, 27] with relatives and problem-centered expert interviews [28] with professionals. The interviews started with a brief naming of the topic. Each interview was followed by an open-ended question designed to invite participants to share their experiences in their own words. To ensure that relevant topics were discussed, interview guides were developed according to Helfferich [29], including the topics of the SELFIE framework [6] (Additional file 2). Follow-up and clarification questions were used to ensure that the content was understood correctly and to allow the interviewee to correct it. Postscripts and brief questionnaires with sociodemographic data supplemented the interviews [26, 27]. The interview guides were each pretested one time. We digitally audio recorded the interviews and they were transcribed by a translational office according to content-semantic transcription [30]. All interviews were intended as individual interviews to encourage participants to speak openly about their experiences.

Next to the interviews, the first author analyzed the files of residents living with dementia. Therefore, a template for data extraction was developed and used to screen the care plans, care reports, orders or faxes. The template included categories for tasks and roles of professional groups as well as topics of the SELFIE framework [6]. During data extraction, all individual passages that related to the underlying definitions of intra- and interprofessional collaboration or that served to understand the tasks of the individual staff groups were documented verbatim from the last six months. The document analysis served to understand, corroborate, and augment the evidence drawn from the interviews or context questionnaires [22].

Context questionnaires that covered care unit and nursing home aspects were used for case descriptions: size, ownership and location of the nursing home and architectural, financial, staff and resident-specific characteristics of the care unit. We further used the Dementia Care Questionnaire (DemCare-Q) [31] to collect data on the application of dementia-specific interventions in the care units.

The data collection process took place from February to August 2022, and all data were collected either in a quiet room in the nursing home or in the physicians’ offices.

### Data analysis

We initially combined all data collected at the case level in a case study database [22]. Regarding quantitative data, we calculated relative and absolute frequencies or means and standard deviations using SPSS Statistics V21 [32] to describe the sample. All other standardized data were analyzed narratively and served to provide case context descriptions and to supplement the qualitative data.

We analyzed all qualitative data with MAXQDA 2022 [33], according to content structuring analysis [34]. By reading the transcripts several times and initiating text work and memos, we first familiarized ourselves with the material. Then, we used a deductive-inductive approach to form main- and subcategories. The main categories were developed theory-based using the interview guide. To code the main categories, the interviews were read line by line. The first two interviews were coded by KS and JD independently of each other. The results were discussed with the project leader (BH). Subsequently, we revised the categories and specified category definitions along discussions. This process was followed by the recoding of three more interviews, further exchanges, and further adjustments. Once there was a common understanding of the main categories, the first author coded all cases. Considering the coding of the main categories, KS inductively developed and defined subcategories for each case. Document passages were either subordinated to or supplemented the categories, and descriptive material from the context questionnaires (context units) was consulted to understand single coding units. This was followed by discussion among the research team and the testing, modification, and application of the revised category system to all qualitative data [34]. To clarify the category definitions and to discuss subjective impressions, JD (Case C) and AF (Case A) also coded one case. This was done independently but with intermediate discussions.

Based on the codes and the descriptive data, we created tabular overviews and case-specific thematic summaries (case descriptions) [22, 34]. We then used the tabular overviews for the cross-case syntheses, as described by Yin, to compare the categories and to search for patterns and differences. This involved contrasting and synthesizing the patterns and themes across the cases so that cross-case characteristics and themes, as well as differences between the single cases, could be identified [22]. Graphics of interrelationships of categories as well as the cross-case differences were finally created to visualize the findings.

### Ethical considerations

We obtained ethical approval from the German Society of Nursing Science (DGP e. V.) (Registration No. 21–032). In accordance with ethical guidelines, participation in the study was voluntary, and written informed consent was obtained from all participants or their legal representatives prior to data collection.

### Rigor

The first author (KS) and the researchers who assisted her with data analysis (JD, AF) are research associates with a nursing-specific master's degree, a nursing education, and experience in different care settings, which provided an understanding of the participants’ explanations. Regular interactions took place with both a methods group and senior researcher of the study team (MR, RP, BH), who supported the process and facilitated discussions of the results. To increase *credibility*, developing and testing of the main categories (KS, JD) and application of the category system was done by two researchers (KS and JD; KS and AF). Moreover, the results of the study and their presentation were discussed in peer groups with researchers from different disciplines and countries. Similarly, the translation of the selected quotes for this article was carried out by the first author and reviewed and corrected by the research team (*reliability*). *Dependability* was achieved through a reflective documentation process in a logbook and the transparent reporting in this article. Furthermore, quotations from the original data were used to allow for an evaluation of the results (*reliability, construct validity*). *Confirmability* was obtained through triangulation between researchers and data sources. To address preconceived notions, the first author also noted her preconceptions prior to data collection and reflected on them as the study progressed (*reliability, construct validity*). For *transferability*, a replication design was used to obtain stable results. Additionally, a thorough description of the research context was provided, allowing readers to interpret the results and decide whether conclusions might be valid in other contexts (*external validity*) [22, 35]. Since this was not an explanatory study, we did not assess aspects of *internal validity* according to the recommendations by Yin [22].

## Results

We selected four care units from four different nursing homes (Table 1), comprising four context questionnaires, 18 resident files of residents living with dementia and 35 interviews with 21 professionals (Table 2) and 14 relatives of residents living with dementia (Table 3).

**Table 1:** Characteristics of nursing homes and care units

	**Case A**	**Case B**	**Case C**	**Case D**
**Nursing Home**				
Ownership	Nonprofit	Nonprofit	Nonprofit	Nonprofit
Number of beds	87	32	63	100
Number of care units	2	2	3	3
**Care Unit**				
Care focus agreed with cost unit	-	Dementia	-	-
**Staff on care unit level**				
RNs 3-year nursing education, FTE	8.00	2.04	6.00	2.75
RNs further training, FTE	3.00	3.65	1.00	2.00
Nursing assistants, FTE	-	2.05	1.35	1.00
Unskilled nurses, FTE	10.00	3.55	4.35	5.25
Nursing trainees, FTE	2.00	2.00	-	3.00
**Residents on care unit level**				
Residents in care unit, n	47	22	26	28
Care level 2, in %	14.90%	-	-	17.86%
Care level 3, in %	42.55%	9.09%	50.00%	57.14%
Care level 4, in %	31.91%	72.73%	23.08%	17.86%
Care level 5, in %	10.64%	18.18%	26.92%	7.14%
Dementia diagnoses, in %	42.55%	100.00%	26.92%	39.29%
**Care and support on care unit level**				
Case conferences	Yes	Yes	Yes	Yes
Pain assessment	Yes, for all	Yes, for most	Yes, for all	Yes, for all
Behavior assessment	-	-	-	-
Dementia severity assessment	-	-	-	-
Quality of life assessment	Yes, for all	Yes, for all	-	-
Depression assessment	-	-	-	-

*RN* Registered Nurses, *FTE* full time equivalent (means full time job positions in the care unit)

**Table 2:** Characteristics of interviewed professionals

	**In Total**	**Care unit A**	**Care unit B**	**Care unit C**	**Care unit D**
**Number of participants**, ***n***	21	5	5	6	5
**Gender**, ***n***					
Female	16	4	3	4	5
Male	5	1	2	2	0
**Age in years**, mean (range)	45.3 (27–65)	43.2 (35–58)	56.4 (47–63)	40.5 (27–56)	42.0 (29–65)
**Professions**, ***n***					
RN (thereof with ST)	14 (11)	3 (3)	3 (2)	5 (5)	3 (1)
Social worker	2	1	0	0	1
Physicians					
General practitioner	2	0	1	0	1
Geriatric-psychiatrist	1	0	1	0	0
Physiotherapist	1	1	0	0	0
Pharmacist	1	0	0	1	0
**Work experience in years**, mean (range)	16.7 (0.5–32)	14.6 (6–20)	25.2 (16–32)	16.3 (8–25)	10.7 (0.5–23)

*RN* Registered Nurses, *ST* specialized training

**Table 3:** Characteristics of interviewed relatives

	**In Total**	**Care unit A**	**Care unit B**	**Care unit C**	**Care unit D**
**Number of participants**, ***n***	14	4	4	3	3
**Gender**, ***n***					
Female	11	4	1	3	3
Male	3	0	3	0	0
**Age in years**, mean (range)	61.2 (36–90)	55.0 (36–63)	64.3 (51–82)	70.0 (53–90)	57.0 (46–63)
**Relationship**, ***n***					
Husband/Wife	1	0	1	0	0
Daughter/Son or Sister/Brother	10	2	3	3	2
Granddaughter/-son	1	1	0	0	0
Niece/Nephew	1	1	0	0	0
Legal guardian	1	0	0	0	1
**Visit frequency**, ***n***					
Daily	1	0	1	0	0
1–2 × a week	10	4	2	2	2
2–3 × a month	3	0	1	1	1

### Case description

**Fig. 1:**
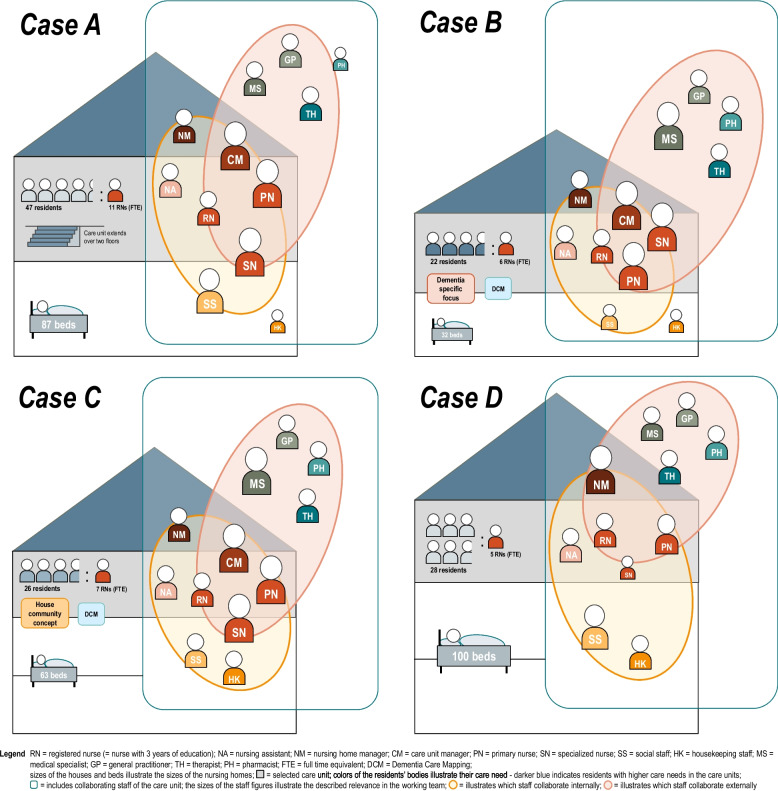
Comparison of the characteristics of the cases

All of the included nursing homes are nonprofit nursing homes located in a semiurban to urban region and include two to three structurally separated care units. The organizational context and current collaboration structure differ in each case (Fig. 1).

*Case A* refers to a nursing home with 87 inpatient beds. The selected care unit comprises 47 residents with mainly medium-level care needs from two residential groups on two floors. Their care is provided by approximately eleven nurses (full time equivalent) with three years of education, three of whom have a specialization in palliative care, mentoring, nursing management, or geriatric-psychiatry. Important roles in coordination and decision-making on site are taken on by primary nurses and the nurse with a specialization in palliative care. The care unit manager plays an important supervisory role in the care evaluation and conducts admission interviews with residents and relatives. Furthermore, nursing and social work are understood to be closely linked, both in care planning and in the provision of support. Next to them, physiotherapists and occupational therapists care for residents as members of external professional groups, and various GPs and one neurologist provide home visits at irregular intervals. Pharmacists and housekeeping staff play a subordinate role in the multidisciplinary team in *Case A*.

*Case B* refers to a small nursing home with 32 inpatient beds. The selected care unit is a dementia-specific care unit with an additional financing regulated by a special agreement. It includes 22 residents with mostly high-level care needs. Their care is provided by approximately six nurses (full time equivalent) with three years of education, two of whom have specialized in palliative care, mentoring, or geriatric-psychiatry. In *Case B*, important roles in evaluation, coordination, and on-site decision-making are taken on by primary nurses, the care unit manager, and the specialized nurse in geriatric-psychiatry. The nursing home manager is consulted in case of uncertainties and conducts admission interviews with residents and relatives. Although nursing and social care are seen as being closely linked in this case, social staff, such as housekeeping staff, play a subordinate role in the multidisciplinary team. Physiotherapists and occupational therapists work with the residents as external professionals. Additionally, various GPs conduct irregular home visits, while a permanent psychiatrist works closely with the care unit and conducts joint visits with the specialized nurse in geriatric-psychiatry at regular intervals.

*Case C* refers to a nursing home with 63 inpatient beds. The selected care unit has an additional financing regulated by a special agreement for a house community concept and includes 26 residents, who mainly have moderate to high-level care needs. Care is provided by approximately seven nurses (full time equivalent) with three years of education, one of whom is a wound expert. In *Case C*, important tasks in evaluation, coordination and decision-making on site are performed by primary nurses, the care unit manager and the specialized nurse in geriatric-psychiatry of the nursing home. The care unit manager primarily assumes control tasks so that all decisions need to be coordinated with him in advance. Nursing, social and housekeeping tasks are seen as being closely interlinked in *Case C*. Physiotherapists and occupational therapists care for residents as members of external professional groups. Additionally, various GPs and neurologists conduct irregular home visits, while one psychiatrist cares for some residents and conducts joint visits with the geriatric-psychiatry nurse at regular intervals. Furthermore, the close cooperation and high value of the pharmacist are characteristic features of *Case C*.

*Case D* refers to a large nursing home with 100 inpatient beds. The selected care unit includes 28 residents with mainly moderate care needs. Their care is provided by approximately five nurses (full time equivalent) with three years of education, two of whom have a specialization in palliative care and mentoring. Specific roles of specialized nurses are not described. The nursing home manager plays an essential role in on-site evaluation, coordination, and decision-making in *Case D*. A care unit manager does not exist. Initial interviews and counseling sessions with residents and relatives are conducted by nurses. In *Case D*, nursing, social and housekeeping workers are seen as important but generally rather independent members of professional groups with clear task delineations. External physiotherapists and an internally employed occupational therapist further care for the residents. Additionally, various GPs make irregular or regular home visits, while residents who receive neurological services are cared for by one neurologist during regular home visits. These visits do not take place together with nurses. Finally, *Case D* is characterized by the fact that the nursing documentation system has thus far not been changed to a digital system.

### Similarities and differences in current professional collaboration

We identified four categories to compare current professional collaboration between the cases: *actors and their roles, service delivery, coordination and governance,* and *communication channel*.

#### Actors and their roles

In all cases, we identified various professional groups as part of the workforce. Even if there are many similarities in their roles, we also identified differences connected to the organizational contexts.

The similarities were found to relate to the basic understanding of most of the roles of nurses, physicians, social staff and therapists; in all cases, *nurses* are identified as providers of body-related care. Therefore, nursing assistants provide basic care, while nurses with three years of education observe changes and are responsible for medical care, and specialist nurses are involved in specific activities such as wound care. Furthermore, nurses with three years of education are assigned the role of coordinators, quality evaluators, and decision makers for internal care processes. *Physicians* are in all cases primarily assigned the role of decision makers for medical orders, prescriptions, or hospitalizations. Moreover, they take on a mediating role by talking to relatives and handling consultations with health insurers. While GPs are responsible for all medications, neurologists or geriatric-psychiatrists focus on behavioral problems and medications in all cases. *Social staff* is attributed an important role in providing and planning group and individual activities and events. Additionally, biography work, the monitoring of preferences and the forwarding of information to nurses and relatives are seen as social care tasks. Among *therapists*, physiotherapists are described as part of the multidisciplinary team in all cases. They ensure that the residents' resources are maintained and that motor skills and quality of life are promoted. *Other professionals*, such as janitors, administrators, cleaners, hairdressers, podiatrists and other medical specialists, are understood to be part of the multidisciplinary team in all cases, although they take on a subordinate role.

Regarding *pharmacists*, the scope of their roles differs greatly between the cases, from providing medications (*Case A*) and advising staff (*Cases B, C*) to taking over medication interaction checks (*Cases B, C, D*). Equally, *housekeeping staff* is barely mentioned in *Cases A* and *B*, while it is attributed a high level of responsibility for nutrition and fluid balancing and is considered important for case discussions in *Cases C* and *D*. Further, *nurses* with three years of education are assigned the role of housekeeping and social services support in *Cases A, B* and *C*, while in *Case D,* these tasks are described only as subordinate tasks of nursing assistants. Similarly, in C*ases A, B* and *C*, specialized nurses are assigned the role of consultants to provide feedback or advice to nurses or students, while this is not the case in *Case D*.

#### Service delivery

In all cases, service delivery in professional collaboration is based on four distinct steps: *recognizing problems and changes, internal decision-making, external decision-making,* and *dealing with discrepancies*. Differences between the cases relate to specific organizational aspects and strategies within the realization of these steps.

In all cases, the most common trigger for collaboration is *recognizing problems or changes* of residents. Here, it is primarily the internal staff who assess such situations. Admission and integration meetings held with relatives and care plans are important information used, including the biographies, preferences and problems of residents living with dementia gathered by nurses, managers, or social staff in consultations with relatives. Everyday observations and conversations with relatives and colleagues then contribute to evaluations of initial situations. Whereas risk assessments and protocols do exist in all cases (Table 1), participants in *Case B* explicitly mentioned that no scales are used in that care unit, but rather the professional opinions.

Based on the identification of problems and changes of residents, an *internal decision-making process* takes place in all cases. Therefore, certain problems are first clarified directly by single persons working on site. In situations that cannot be decided alone, an exchange within the team is considered important in all cases. This involves discussions about observations and different views or experiences. If necessary, the team consults nurses from other care units or the management. Measures are then jointly defined on the basis of this exchange within the nursing home in all cases.

“So I wouldn't decide on my own now. I could, but I wouldn't, because we can simply advise each other as a team.” (Case A, professional 1, line 41)

Differences in the realization of internal decision-making arise from the hierarchy of the nurses in the cases; while the primary nurse plays an important role in case discussions in *Case A* and makes decisions after the team exchange, *Case B* describes a stronger joint decision-making and in-house opinion formation, together with the nursing home management. In *Cases C* and *D*, in contrast, it is emphasized that the primary nurse cannot decide anything without consulting the care unit manager (*Case C*) or nursing home manager (*Case D*), who ultimately decides. Further, the integration of social and housekeeping staff differs across the cases, from exchanging new information with social staff (*Cases A, D*) to joint discussions and care planning with social (*Cases A, B*) and housekeeping staff (*Cases C, D*).

If changes linked to the resident care cannot be decided internally, an *external decision process* is initiated. In all cases, this starts with an exchange with external professionals. This includes the pure forwarding of information or a joint situation assessment, in which problems are addressed, therapy successes are evaluated and adjustments are discussed. The joint situation assessment between nurses and GPs is described in all cases. Besides that, there is a special focus on joint assessments made between the geriatric-psychiatrist and the specialized geriatric-psychiatry nurses in *Cases B* and *C*. Further, joint situation assessments between nurses and physiotherapists (*Cases A, B*) or pharmacist (*Case C)* are described in some of the cases. The first exchange is followed by joint action planning. Here, joint action planning with physicians is described most frequently but to a very different extent in all cases. A common feature is that suggestions are initially formulated by nurses on the basis of their experiences and observations. However, although in all cases it is emphasized that physicians decide together with nurses and predominantly respond well to suggestions, the final decision rests with the physician in all cases.

Finally, different strategies are used in *dealing with discrepancies*. The strategy of argumentation and discussion to reach a compromise is described in all cases and by all professionals. In *Cases A*, *C* and *D*, the nurses also describe the strategy of influencing the decision through the input that is passed on. Other strategies include bypassing the person who shares a different opinion or reports back only very sporadically and, for example, directly contacting the specialist instead of the GP (*Case D*) or changing the physician (*Cases A, B, D*). Further, instrumentalization of relatives is also used (*Cases A, C, D*), as relatives confront the physicians differently and their demands are often answered more quickly.

"I discussed with the care unit manager that I would like to ask the physician again myself. We agreed […] and then I went to the physician, discussed it and passed on the information here." (Case C, relative 9, line 81).

If there are no discrepancies and relatives’ consent is not needed, relatives are not involved in decision-making and get only informed after an event or if they proactively involve themselves in all cases.

#### Coordination and governance

Concerning coordination and governance, similar aspects of *internal and external coordination* are distinguished in all cases. Differences between the cases refer to the named coordinating nurse and the influence of the care complexity.

In all cases, *internal care coordination* refers primarily to the delegation and organization of staff. Nurses with three years of education are responsible for organizing daily care according to priorities and for delegating tasks to nursing assistants. The decision of whether a nurse with three years of education or a nursing assistant is responsible for a resident is based on the individually perceived care complexity.

“We assess whether it still fits that a resident is included in the assistant tour because, due to nonexistent medication, something can happen at any time, so you have to react immediately, also medically.” (Case B, professional 10, lines 135–137).

Similarly, social staff and nursing assistants divide up who takes on which activity program depending on their further training and experience in all cases.

For *external care coordination*, nurses are particularly responsible for information sharing. This includes telephone calls with physicians and relatives; information transfer between physicians; communications with social staff, management, pharmacy, medical stores and janitors; and organizing physiotherapy. As they are the only professionals that are in contact with every other profession, nurses are responsible for coordinating overall care with all information coming together in the duty room. In addition to nurses, GPs also identify themselves as coordinators and main contact points for treatment care in *Cases B* and* D*, as they assess the overall care situation and decide on changes, even if they do not have direct contact with other professionals and obtain most of their information through nurses.

The specific nurse who ultimately assumes the coordinating tasks varies according to *availability* due to shift work, vacation, and illness in all cases. Furthermore, *care complexity* is perceived as an influencing factor in some cases. Although primary nurses are assigned to residents and responsible for internal care planning and evaluation, as well as external information sharing in *Cases A, B* and *C*, more joint coordination takes place in *Cases B* and *C* if residents have a higher need for observation. Likewise, *Case A* describes a stronger support of the care unit manager if a primary nurse is unable to coordinate situations. In contrast, in *Case D*, no adjustments in coordination corresponding to complexity are identified. This could be due to the already high relevance of the nursing home manager in the process of coordination, evaluation and decision-making.

#### Communication channel

Regarding communication channels, we identified similar *channels for information sharing* in all cases. Differences between the cases are related to *interactive information exchange*.

Important *information is shared* internally by e-mail and tabs in the resident files. Further, protocols document the progress and decisions made to enable evaluations and to inform people not involved in the process. Similarly, prescriptions and diagnostic reports, as well as telephone calls, fax or e-mails, are used for external information transfer in all cases.

Besides that, *interactive information exchange* serves to jointly assess situations and discuss different point of views. For this purpose, internal team handovers are used in all cases to enable an exchange within the nursing team (*Cases B, C*) or between nursing and social staff (*Cases A, D*). Additionally, nursing home visits by physicians are described as possibilities for exchange with external professional groups. Therefore, there are differences described between regular, sporadic and emergency visits according to the preferences of different physicians in all cases. While it is emphasized in all cases that some of the physicians would not carry out nursing home visits alone, others simply communicate their decisions afterward without having any exchange with nurses (for example, neurologist in *Case D*).

Furthermore, case conferences and team meetings are established structures in all cases. In these, the focus is either on talking about residents on a case-by-case basis (*Cases A, B*) or on talking about the care unit, current problems and possibilities for improvement on a topic-by-topic basis (*Cases C, D*). These meetings are carried out within the nursing team, with managers and relatives or internally interprofessionally with social (*Cases A, B*) and housekeeping staff (*Cases C, D*). Specialized nurses from other care units and external professionals do not participate in case conferences. Exceptions were unique situations in which physicians participated in *Cases B* and* C*.

“I [as a physician] have already experienced [team meetings] two or three times, […] when there were completely different opinions of relatives […]. But that happens maybe once a year.” (Case B, professional 9, line 83).

Finally, situational exchanges in the duty room or corridors are described as opportunities to exchange information. In particular, the exchanges with or between therapists are described as situational and random in all cases. Likewise, situational exchanges between nurses and social staff (*Case B*), as well as those between social staff and physicians (*Case D*), are mentioned.

### Similarities and differences in strengths and problems of professional collaboration

Three categories were identified relating to strengths or problems of professional collaboration: *role understanding, teamwork,* and *communication and exchange*. Although we identified the strengths and problems in all cases, more strengths in teamwork and communication were named in *Cases A* and* B*, while in *Cases C* and* D,* more problems in role understanding, teamwork and communication were specified.

#### Role understanding

Regarding role understanding, clear structures and distribution of tasks are seen as an existing strength. In all cases, the clearly separated responsibilities of nurses, social and housekeeping staff and nurses with different qualifications are mentioned positively. In contrast, overlaps in internal tasks (*Cases C, D)*, the lack of a main contact person (*all cases*) and the proactive approach of relatives directly contacting the physician without talking to nurses are perceived as problematic for a clear role understanding. Similarly, role ambiguities between external professional groups are identified as problematic, as responsibilities for medication monitoring (physicians and pharmacists) or treatment orders become blurred (GPs and medical specialists). This leads to dissatisfaction and missing orders or premature orders of medications by other physicians.

"No physician feels responsible for prescribing physiotherapy or occupational therapy. The GP says ‘He has dementia, I don't have a diagnosis to write that out, you have to ask the neurologist’, […] neurologist says, ‘I don’t have a diagnosis for that’, so this resident does not receive therapy." (Case D, professional 17, line 38).

Nevertheless, clear distributions of tasks are also perceived as problematic if these clear delimitations reduce the support between professional groups (*Case D*).

#### Teamwork

*Mutual support* is seen as a strength of teamwork. It is considered positive that caring for complex residents with, for example, aggressive behavior or tendencies to leave the nursing home unseen is undertaken as a team. Therefore, either care tours are changed or joint approaches are tested. Likewise, social staff provide one-on-one care to ease the burden placed on nurses (*Cases A, B*), and nurses help social staff at events (*Case D*). Further, residents are motivated by nurses to participate in physiotherapy (*Case A*) and pharmacists asks physicians for prescriptions to support nurses on site (*Case C*). Next, *mutual trust and openness* are perceived as positive in all cases. These factors refer to having trust in nursing and social staff, as well as to openness to different opinions within discourses with internal or external professionals (*Cases A, B*). For mutual trust between physicians and nurses, the length of collaboration, the recognition of close contact between nurses and residents and the awareness of different professional opinions are perceived as supporting factors. In all cases, however, this mutual trust and openness depends on the individuals.

*Mutual dependencies*, on the other hand, are supported by given structures in all cases. Nurses are dependent on information provided by social staff, nursing assistants and relatives, and must consult the management (*Cases B, C, D*) or physicians (*all cases*) before making decisions. This is seen as particularly critical when there is no reaction from the professionals on whom one is dependent, when the entire internal team agrees on necessary measures and yet remain dependent on someone, or when these decisions lie within the competence of one’s own profession.

"I think it is just annoying when you have basically identified the problem quite clearly, but then you have to rely on the physician to make it happen […]; we have to get things signed off on ourselves that relatives can bring in over-the-counter." (Case C, professional 12, lines 358–394).

Other problems related to teamwork are described as being due to *crossing professional groups*. This problem is described in *Cases A*, *C* and *D* and is perceived as existing and problematic by all professions equally. Physicians criticize nurses for bypassing them by involving other professionals who make decisions that run counter to their own ideas. Likewise, social staff, nurses, and physiotherapists feel ignored when they are not taken seriously in their assessments by other professionals or relatives. Results of the dependencies and the crossing of professional groups can be a threat to resident care and dissatisfaction among professionals and relatives.

#### Communication and exchange

Complete *forwarding of information and documentation* is described in all cases as an existing strength. Accordingly, protocols are described as a good tool for passing on information and concrete agreements, and fast reactions are helpful for effective communication. In contrast, it is described as problematic that nursing reports are not always well kept or that information is often communicated verbally and/or inadequately by nursing assistants, social staff or even relatives. This can lead to information being lost. Equally problematic is the inadequate forwarding of information, which contributes to problems not being properly assessed and duplicate inquiries becoming necessary, resulting in unnecessary time wastage. Similarly, arrangements that work are also perceived by relatives as providing relief, while coordination problems or a lack of information forwarding are perceived as problematic. These include situations in which important changes, such as the closure of a medical practice (*Case B*), or the absence of physiotherapy (*Case C*), which require the relatives' intervention, are communicated only by chance or on request.

Existing *internal team and case discussions* are rated as important and helpful (*Cases B, C, D*). They are seen as opportunities for open communication and as a medium for making arrangements with relatives. However, such structures are often criticized; e.g., case conferences either do not take place regularly (*Case D*), or without external moderation (*Case C),* and not all residents can be discussed every time (*Case B*). Further, there are no handovers between nursing and social staff (*Case B*), or within the social team (*Case C*).

With regard to *exchanges with external professionals*, joint visit rounds are rated as positive in all cases. However, the fact that some physicians do not conduct visits in the nursing homes or perform unannounced visits is described as a structural problem in all cases. Additionally, the fact that external professional groups such as pharmacists, physiotherapists and physicians do not participate in case discussions and that there are no joint documentation systems with external professional groups is criticized. Further, there are no structures for exchanges between different physicians in all cases. Missing exchanges and the inadequate care of residents can be related consequences of such a situation.

While in *Case D*, one interviewee describes easy *accessibility* of physicians, all other interviewees, as well as the residents’ files, describe the accessibility of physicians and other external professionals as a problem that has intensified since the coronavirus pandemic. Physiotherapists are seen as overbooked, and Mondays and Fridays, as well as holidays and weekends, are considered problematic days for reaching GPs or other medical specialists.

“Mrs. M did not receive physiotherapy because the prescription had expired. However, there is no physiotherapy practice that can take over Mrs. M. An intensified search was made for physiotherapy practices.” (Case C, resident file 11).

This increases the organizational workload of nurses and leads, for example, to materials or therapy not being requested in time or to unnecessary hospital admissions.

## Discussion

We revealed four categories that describe current intra- and interprofessional collaboration in German nursing homes: *actors and their roles, service delivery, coordination and governance,* and *communication channel*. Moreover, we identified three categories related to the strengths and problems of current collaboration: *role understanding, teamwork,* and *communication and exchange*. These categories provide valuable information about how intra- and interprofessional care is currently organized and what problems and strengths are associated. Furthermore, they highlight both similarities and differences between the cases. In the following, we discuss similarities between the cases and key differences.

### Similarities between the cases

We identified a variety of professionals as relevant members of the multidisciplinary team with more or less demarcated roles. Complementing previous studies [5, 15, 17], these professionals included not only nurses, physicians and therapists but also social workers, housekeeping staff, and pharmacists. Similar to the findings of Tsakitzidis and colleagues, the tasks and roles of different actors were described to be rather separate from each other [15]. This illustrates that current intersections were considered critical. Building on this, ambiguous role and task profiles were described as problematic in our results, as they have been in German and international studies in both interprofessional collaboration [15, 17] and intraprofessional collaboration [36]. Furthermore, these role ambiguities contradict the WHO's definition of collaboration in practice, which is based on the perception of others and their roles [8]. As role ambiguities have been described as problematic for the success of nurse-led care models [5], it is needed to better understand what constitutes these ambiguities in future interventions to improve collaboration.

Although a shared vision of achieving the best possible care for residents was described, collaboration with external professionals was further shown to be fragmented, lacking shared accountability and not being on an equal footing. This is consistent with the findings of Tsakitzidis and colleagues [15]. Similar to other studies [37], aspects of mutual support of different professions were perceived as facilitating factors for job satisfaction but were only rarely described. In particular, collaboration with external professionals was found to occur more likely at the necessary level of interdependence [9]. Possibly, this is reinforced in Germany by the existing hierarchical asymmetries: while nurses in other countries can take over medical tasks independently [38–40], task distribution in Germany is based on delegation by physicians, which means that nurses are not allowed to decide about non-prescribed medications. The effects could be that shared decision-making processes are considered less necessary since physicians (GPs and specialists) alone are liable for these decisions. However, some of these experienced hierarchical asymmetries might change over the next few years due to a new law that will allow academically trained nurses to implement substituted tasks (e.g., in dementia care) from 2025 onward that were previously the responsibility of physicians (BGBl. 2023 I No. 359). To enable this new level of collaboration in the near future, it will be necessary that effective teamwork, mutual trust and openness do not depend on the collaborating individual; rather, working together should be strengthened in general through responsibility transfers and communication at equal levels [15, 37] to address the current problems of low levels of mutual respect.

Regarding internal coordination, our results showed that care is normally divided according to complexity and coordinated internally by one nurse. External coordination was described only selectively. Although nurses were most often attributed the role of coordinators by all professionals, they were not named leaders of the external team. However, documentation of a team leader was identified as an important component of successful interdisciplinary interventions [10]. Further, our results showed, that the nursing tasks in interprofessional coordination primarily relate to information sharing. Leadership tasks were not described in any of our cases. An international review has shown that nursing leadership in residential aged care facilities is associated with high-quality care; nonetheless, the authors also criticized that nurses in geriatric care have few opportunities to acquire confidence and leadership skills, as there is no access to leadership training specific to geriatric care [41]. This situation currently also limits the opportunities for nurse-led care in Germany and should be considered in future intervention studies.

Finally, we identified problems regarding internal and external forwarding of information and direct exchange. While the exchange of information within the internal team comprised comparable levels, as described in other studies [16], exchange platforms with external professionals were missed. This is in line with the results of German studies on collaboration between GPs and nurses during nursing home visits [17, 42]. Regarding the reduction in behaviors [10] and medication prescriptions [43], however, formal team or case conferences were found to be important components of successful interdisciplinary interventions in nursing homes internationally. Similarly, the more active involvement of GPs and pharmacists in interprofessional teams was associated with positive outcomes [10, 43]. Contrary to other studies, in which GPs' choices to make visits were seen as dependent on distances from nursing homes and relationships with residents [15], our results showed that this willingness seems to be more dependent on the value that individual physicians generally ascribe to nursing home visits. Accordingly, future interventions should address the motivation of involved physicians. Relatedly, Resnick recommended reimbursement mechanisms that could contribute to the realistic participation of physicians in team-based activities [44]. Next, our results emphasized the focus on collaborative physicians – positively endorsed to the nursing home. This idea is in line with other German studies [17, 42] and could be associated internationally with better communication and higher levels of trust and recognition [45]. For regular exchange with therapists or pharmacists, we could not identify any structures, although the latter wished to be more involved. This leads to only few contact points between internal and external professionals in decision-making and should be addressed in future intervention developments.

### Key differences between the cases

According to a typology of care units in German nursing homes [46], our selected cases can be most closely assigned to the ideal types of "usual segregated care units" (*Cases A, C, D*) and "dementia special care units" (*Case B*). Nevertheless, despite the in part very similar characteristics of the cases, they differ strongly in their organizational contexts and current collaboration structures.

Differences in collaboration between the cases were based on fundamentally different understandings of task distributions. While for example, in some cases, housekeeping staff were firmly involved in case conferences, they were not seen as part of the internal team in other cases. Additionally, the care units showed different management structures (more vs. less controlled; management as team member vs. as a decision-making authority) that lead to different internal regularities. Differences further emerged in external collaboration, as specialists such as geriatric-psychiatrists were strongly present in some cases, while neurologists played a minor role in other cases. Moreover, the extent of strengths and problems described varied according to the contexts and individuals. This phenomenon is consistent with the findings of other qualitative studies, in which interview quotes have indicated that physicians describe some nursing homes as *"better nursing homes"*, because the task distributions are clearer and collaboration is more structured [15]. Overall, these results illustrate that care collaboration differs relevantly according to the context. It is a well-known recommendation to adapt complex interventions to the heterogeneity of usual care considering the respective context [47, 48] and to describe usual care more concretely [49]. Besides that, relevant context-specific differences in usual care collaboration should be addressed in the development of new care models.

### Strengths and limitations

The case study design provided an in-depth investigation and description of current collaboration in nursing homes. By comparing several cases, we were able to present similarities and differences in nursing homes that have not yet been investigated in this format. A limitation of the results is the sampling. Because professionals and relatives were recruited by the nursing homes, it is possible that more satisfied relatives and/or rather positive-minded professionals were contacted. However, it is also possible that, regarding relatives, those who were very critical were more likely to respond to the research team. Both of these factors may have influenced the results. Further, the professional sample was based on a very large proportion of nurses. This was mainly because there was a particularly high level of interest in this group to participate. This allowed us to focus more on nurses, whose job satisfaction is of particular importance for developing a nurse-led care model. Nevertheless, this fact should be considered when interpreting the results. Due to a lack of willingness to participate, it was also not possible to include every profession in each case. This may have resulted in the focus varying between the cases. We further did not include observations in our data collection, as this would have been very time-consuming (multitude of situational collaboration). However, observational data might extend our results and should be considered in future research. Finally, we did not interview residents living with dementia based on previous studies [17]. Instead, they were included in a second part of the study that focused on dementia-specific care.

## Conclusion

This study provided in-depth insight into intra- and interprofessional collaboration in the care of residents living with dementia in German nursing homes. Four categories describing intra- and interprofessional collaboration (*actors and their roles; service delivery; coordination and governance; communication channel*) as well as three existing strengths and problems (*role understanding; teamwork; communication and exchange*) could be identified. Overall, the results showed that current collaboration – especially with external professional groups – is still quite fragmented and based on little teamwork. Contrary to the definition of collaboration itself, role ambiguities, including a lack of a named leader and clear leadership roles, do exist. Further, external decision-making remains focused on nurses and GPs, not including other important professionals. These problems should be addressed in practice and research. The cross-case analysis additionally highlighted the importance of internal and external structures as well as individuals, influencing current collaboration through fundamental different regulations and understandings. Therefore, our description of current collaboration and associated strengths and problems can enable general implications for future intervention development and evaluation, as well as central and context-specific collaboration aspects for a new nurse-led care model.

### Supplementary Information

Additional file 1. Reporting guideline for organizational case studies.

Additional file 2. Interview guidelines.

## Data Availability

The datasets used and/or analyzed during the current study are available from the corresponding author on reasonable request.

## Abbreviations

DemCare-Q: Dementia Care Questionnaire
DSS: Dementia Screening Scale
GP: General practitioner
SELFIE: Sustainable intEgrated chronic care modeLs for multi-morbidity: delivery, FInancing, and performancE
WHO: World Health Organization
