# Supplementary material for: Current situation, strengths and problems in intra- and interprofessional collaboration in German nursing homes – A holistic multiple case study
Source: BMC Geriatr. 2024 Jul 17;24:610. doi: 10.1186/s12877-024-05182-z (PMC11253382; doi:10.1186/s12877-024-05182-z)
Supplement: Supplementary file 1 — Additional file 1. Reporting guideline for organizational case studies. [file 12877_2024_5182_MOESM1_ESM.pdf]

Additional file 1

Consensus standards for the reporting of organizational case studies

Reporting item		Page number on which item was reported	Page number of justification for not reporting
Describing the design			
1	Define the research as a case study	Titel; Abstract; 3	
2	State the broad aims of the study	3	
3	State the research question(s)/hypotheses	3	
4	Identify the specific case(s) and justify the selection	3	
Describing the data collection			
5	Describe how data were collected	4	
6	Describe the sources of evidence used	4	
7	Describe any ethical considerations and obtainment of relevant approvals, access and permissions	5	
Describing the data analysis			
8	Describe the analysis methods	4-5	
Interpreting the results			
9	Describe any inherent shortcomings in the design and analysis and how these might have influenced the findings	14	
10	Consider the appropriateness of methods used for the question and subject matter and why it was that qualitative methods were appropriate	14	
11	Discuss the data analysis	14	
12	Ensure that the assertions are sound, neither over- nor under-interpreting the data	12-14	
13	State any caveats about the study	14	

Rodgers, M., Thomas, S., Harden, M., Parker, G., Street, A., & Eastwood, A. (2016). Developing a methodological framework for organisational case studies: a rapid review and consensus development process. *Health Services and Delivery Research*, *4*(1), 1-170. https://doi.org/10.3310/hsdr04010
