# Supplementary material for: Current situation, strengths and problems in intra- and interprofessional collaboration in German nursing homes – A holistic multiple case study
Source: BMC Geriatr. 2024 Jul 17;24:610. doi: 10.1186/s12877-024-05182-z (PMC11253382; doi:10.1186/s12877-024-05182-z)
Supplement: Supplementary file 2 — Additional file 2. Interview guidelines. [file 12877_2024_5182_MOESM2_ESM.pdf]

Interview guide – professionals

Topic	Questions
**Interview aim**	**Brief introduction:** We would like to develop an idea of what a care model in residential long-term care could look like - to this end, we would like to reflect with you how care of residents living with dementia is currently provided here in the care unit. We focus on professional collaboration between the different professional groups.
**Introduction**	**Please imagine a resident living with dementia who lives here in the care unit.**<br>How does professional collaboration currently take place in the care of this resident?
**Actors and their tasks and roles**	**Which professional groups are involved in the care of this resident and what tasks or roles do they assume?**<br>To what extent are these tasks and roles clearly defined?<br>Is this different for other residents? What is different there?
Own tasks/ role	**Where do you see your own tasks and your own role in the care of this resident?**<br>For which tasks do you feel responsible?<br>What skills and competencies do you think you need to perform these tasks?
**Service delivery**	**Could you please describe how care decisions are made for this resident?**<br>How is the resident's care situation [care problems] assessed?<br>How is it decided that something should be changed in the resident's care?<br>Who is involved in decision-making/care planning?<br>How are disagreements between professional groups handled?
**Leadership and governance**	**Can you describe how care for this resident is coordinated between the various professional groups?**<br>Is there one person who is responsible for coordination? Who is this person and what are his/her responsibilities?<br>How is it handled when the resident's care needs become more complex?
**Communication**	**How do the professional groups who are responsible for this resident communicate with each other?**<br>How are important information about the resident communicated within and between the professional groups?
**Problems and strengths in collaboration**	**Have there been situations with this resident, in which care was not provided appropriately?**<br>Can you describe these situations? What were the problems?<br>What would have helped?<br>What were the outcomes of these situations?<br>**Were there situations where care could not be delivered/coordinated quickly enough by the professional groups involved?**<br>Can you describe these situations? What contributed to these situations?<br>What could have gone better in these situations?<br>What were the outcomes of these situations?<br>**Are there also examples in which the collaboration took place very well?**<br>What were positive aspects?
**Measures of collaboration**	**Are there specific measures that are already being taken to promote collaboration or exchange between professional groups?**<br>How are these measures designed?<br>How do you feel about these measures?<br>What kind of needs do you see?
**Problems and strengths in general**	**What strengths and problems do you currently perceive in care overall? Can you tell me about them?**<br>What is currently working very well? What should remain as it is?

	What is working less well? Where are problems?<br>What strengths and weaknesses do you perceive in professional collaboration?
**Vision of care**	**If care in the care unit would meet your expectations, what would good care look like?**<br>What does "good" care mean to you?<br>What would be your roles and tasks?
Changes needed	**In your opinion, to what extent does the current care fulfill this expectation? What changes do you think are necessary to achieve this?**<br>What would be the most important goal of change?<br>What would be additionally desirable but less important?
**Closing**	**Are there any other things you'd like to mention on the topic that we haven't addressed so far?**

Interview guide – relatives

Topic	Questions
**Introduction**	**I would like to know more about how your relative is receiving care and being cared for here in this care unit. I would like you to tell me about the care from your point of view.**<br>What does a typical day look like?<br>What do you consider to be part of everyday care?
**Actors and their tasks and roles**	**Which professional groups are involved in the care of your relative? What tasks and roles do they assume?**<br>How do you experience the collaboration between the different professional groups?<br>In your opinion, how does the collaboration between the individuals work?
**Problems and strengths in general**	**Please tell me three aspects of care in the care unit that you think work really well.**<br>Are there other things that you are currently very satisfied with?<br>Which other things should not change?<br>**Which three aspects are working less well?**<br>What do you think is the biggest problem?<br>What are other problems?<br>Where do you see problems in collaboration?
**Service delivery and communication**	**Do you feel that your relative is involved in care planning or decision-making? Can you elaborate on that?**<br>**To what extent are you yourself involved in care and its planning?**<br>Who talks to you about your relative's problems or acute symptoms?<br>Who informs you about decisions made? Who discusses them with you?<br>How are different views dealt with?
**Coordination**	**If you now think of specific situations here in the care unit in which your relative acutely needed help. Can you describe these situations?**<br>Who did you talk to then?<br>What happened then?<br>What could have gone better in these situations?
**Problems and strengths in collaboration**	**Were there also situations in which your relative could not be helped quickly enough?**<br>Can you describe these situations?<br>What do you think contributed to the fact that help was not given quickly enough?<br>What happened next?<br>What do you think would have helped?

	**Sometimes there are problems in communication between relatives and staff. Were there situations in which you yourself, as a relative, did not feel heard or understood?**<br>Can you give me an example?<br>What would you have wished for?<br>**Were there also situations in which the collaboration worked very well? Can you give me an example?**
**Vision of care**	**Finally, if you imagine that the care here in the care unit would meet your expectations. What would good care look like?**<br>What does "good" care mean to you in this case?<br>To what extent does the current care fulfill this in your opinion?<br>How would the collaboration have to be designed from your point of view?
Changes needed	**What changes in the design of care would you like to see?**<br>What would be the most important goal of change?<br>What would be additionally desirable but less important?<br>What should not be changed?
**Closing**	**Are there any other things you'd like to mention on the topic that we haven't addressed so far?**
